# C-Norm: a neural approach to few-shot entity normalization

**DOI:** 10.1186/s12859-020-03886-8

**Published:** 2020-12-29

**Authors:** Arnaud Ferré, Louise Deléger, Robert Bossy, Pierre Zweigenbaum, Claire Nédellec

**Affiliations:** 1 Université Paris-Saclay, INRAE, MaIAGE, Jouy-en-Josas, France; 2 Université Paris-Saclay, CNRS, LIMSI, Orsay, France

**Keywords:** Entity normalization, Neural networks, Ontology, Few-shot learning, Vector space model

## Abstract

**Background:**

Entity normalization is an important information extraction task which has gained renewed attention in the last decade, particularly in the biomedical and life science domains. In these domains, and more generally in all specialized domains, this task is still challenging for the latest machine learning-based approaches, which have difficulty handling highly multi-class and few-shot learning problems. To address this issue, we propose C-Norm, a new neural approach which synergistically combines standard and weak supervision, ontological knowledge integration and distributional semantics.

**Results:**

Our approach greatly outperforms all methods evaluated on the Bacteria Biotope datasets of BioNLP Open Shared Tasks 2019, without integrating any manually-designed domain-specific rules.

**Conclusions:**

Our results show that relatively shallow neural network methods can perform well in domains that present highly multi-class and few-shot learning problems.

## Background

### Introduction

A huge amount of information is spread in the scientific literature, particularly in the life science and health domains. Information extraction methods aim to extract this information to build or enrich knowledge bases. Yet, natural language is highly variable and a single semantic entity can be expressed in different forms in text (e.g. synonyms or hyponyms). Entity normalization (also called entity disambiguation, entity grounding, or entity linking) is an important subtask of information extraction that addresses this issue by linking entity mentions in text to categories or concepts of a reference vocabulary. In Life Science and Health domains, entity normalization allows textual mentions to be grounded with the same references as databases (e.g. sequence banks, disease control records), improving data interoperability.

Normalization can be seen as a multi-class classification problem where the entities are the examples to be classified and the categories or concepts are the classes. The reference vocabularies can be formal semantic resources such as ontologies [1, 2]. An ontology concept can be labelled by several terms (e.g. synonyms). Each concept can be used as a semantic reference for textual entity mentions. For instance, the concept identified by OBT:001623 in the OntoBiotope ontology [3, 4], and labelled by the term “*lymphocyte*”, could normalize textual entity mentions such as “*lymphocytes*”, “*lymphocytic*”, “*t-cell*” or “*monoclonal B cells*”.

In specialized domains, this classification problem is frequently characterized by a large number of classes but few manually annotated training examples. It results in a highly multi-class and few-shot learning problem [5, 6] and even a zero-shot learning problem [7], namely a problem where some predictable classes have no positive example in the available training set. Few-shot learning is a well-known challenge for machine learning, which remains mainly addressed by weakly supervised and transfer learning approaches.

In this article, we propose C-Norm (“Concept-NORMalization”), a new shallow neural method to address the few-shot learning normalization problem. C-Norm synergistically combines standard and weak supervision, ontological knowledge and distributional semantics. We assessed the method on the Bacteria Biotope normalization (BB-norm) task [7] of the BioNLP Open Shared Tasks (BioNLP-OST) 2019 [8]. This task aims to normalize mentions of microbial habitats and phenotypes with concepts from the OntoBiotope ontology, which are expressed in variable forms in text. C-Norm outperforms existing methods evaluated on the BB-norm dataset, without using manually-designed and domain-specific rules and with low requirements of computational resources compared to large neural networks.

### Related work

#### Pattern-matching rule-based methods

Classic strategies to normalize textual entities rely on the similarity between entity forms and concept labels [8]. Due to frequent linguistic variations (e.g. noun-phrase inversion, typographic variations, synonymy), these methods are dependent on comprehensive lexicons. Several strategies are used to ensure comprehensiveness: third-party resources [9, 10], inflection generation [11–13], pre-processing (lemmatization, stemming or stopword filtering) [14], giving more weight to syntactic heads of mentions and labels [15]. These methods are commonly limited to a given domain/task because they depend heavily on domain-specific resources (e.g. involving specific blacklists, specific disambiguation rules), which results in a poor adaptability.

#### Static word vector-based methods

Vector representations of words enable to handle textual entities in a formal representation. Ranging from TF-IDF bag-of-words [16] to embeddings [17–20], these word representations are largely used in recent natural language processing systems, especially in combination with machine learning systems. For entity normalization tasks, they are mainly used to compute a similarity measure in the same vector space between representations of text mentions and concept labels. For each mention, the concept with the most similar label is predicted. Machine learning is not required beyond a simple 1-nearest-neighbor algorithm: a cosine similarity measure can be used to detect form similarities between both expressions [21, 22] or to handle linguistic variations [23]. However, these approaches have limitations. TF-IDF bag-of-words representations fail to detect similarities between expressions which share no common words. Word embeddings, despite their strength, do not fully succeed to account for domain-specific meanings [24] without integrating some external knowledge [25].

#### Machine-learning and vector-based methods

Manually produced examples, such as couples of mentions to normalize and concepts, provide useful knowledge about a task and domain. By using them, machine learning (ML) algorithms aim to predict concepts from input mentions that exhibit similar features to the training examples. The goal of such methods is to adapt to a task and domain just by using associated examples. Adaptability is what makes ML methods competitive in many tasks, as long as there are enough good-quality examples available for the target task.

ML algorithms mostly use vectors to represent inputs and outputs. They learn to transform input representations of text mentions in a way that optimizes the similarity between the final transformed vectors of mentions and the vectors of their associated concepts.

Historically, D-Norm [26] was the first method which addressed the normalization problem in this way, by using TF-IDF bag-of-words representations for input mentions and for concept labels. D-Norm also overcomes the limitations of TF-IDF bag-of-words by learning a linear projection of the representations of mentions into the space composed by the representations of the labels of concepts. The linear projection must maximize the dot product between mention vectors and label vectors from the associated concept, even for mentions and labels with no common token. Nevertheless, TF-IDF bag-of-words are high-dimensional, sparse vectors, which operate poorly with machine learning algorithms.

The CONTES method [27] addresses normalization in a way similar to D-Norm. However, instead of TF-IDF bag-of-words, it represents vectors of entity mentions by averaging the word embeddings of the words composing the mentions, and it represents vectors of concepts by relying on the hierarchical structure of the reference ontology, without using the ontology labels themselves. The method uses word embeddings as input, which overcomes the limitation of TF-IDF bag-of-words representations. CONTES uses the ordinary least squares method to estimate the linear projection parameters, which struggles to scale up on large training datasets and large ontologies.

In the general domain, the state-of-the-art methods to address entity normalization in that way are large neural nets [28]. The reason may hold in their capacity to model hard nonlinear phenomena. From a computational point of view, neural networks have the asset of being online algorithms. Nevertheless, large neural nets seem to have difficulty addressing the few-shot learning problem. In fact, in specialized domains, shallow convolutional neural networks (CNNs) have been used with some success in normalization tasks with small training data. Their purpose is to calculate an intermediate representation of an expression from the embeddings of their tokens [29], or to detect specific tokens (or contiguous sequences of tokens) that could trigger a specific class [30]. There still seems to be considerable room for improvement.

#### Sieve-based and ensemble approaches

In order to improve performance, another strategy is to integrate several methods (i.e. as components) at once to take advantage of their complementarity. For instance, rule-based methods can make predictions even in a zero-shot learning context and frequently achieve good accuracy, but poor recall (i.e., they cannot make predictions for all mentions). In contrast, vector-based machine learning methods achieve better recall. Thus, a common sieve strategy is to first use a method with a high accuracy, preserve the predictions and pass the mentions without prediction (or the mentions with predictions estimated as uncertain) onto another method [29, 31]. A limitation of combining methods in this pipelined way is that the second method will not get the opportunity to make a prediction for every entity and prediction errors from the first method are propagated.

Ensemble approaches are a more synergistic way of combining multiple components. They are considered the state-of-the-art for many machine learning problems [32]. The advantages of ensemble methods lie in their higher expressive power compared to their single components, as well as in a reduced risk of overfitting. And yet, to our knowledge, ensemble methods are still surprisingly uncommon for entity normalization in specialized domains. An example is the method of Deng et al*.* [29] which follows a voting ensemble strategy where several identical neural components are trained on the same example set, but the random initialization of each component results sometimes in different predictions. Then, for each mention, the concept predicted by a majority of models is chosen.

In this paper, we build on previous work and combine two vector-based neural methods in an attempt to overcome their limitations. We design a new method that integrates the two approaches in an ensemble averaging way and compare its performance to a more rigid sieve-based approach. We also compare it to several existing approaches, including the ensemble-based method of Deng et al*.* [29].

## Methods

In this section, we give an overview of the normalization task we are addressing. Then we describe our methods, as well as our experimental setting.

### Bacteria Biotope dataset and task

We used the *BB-norm* dataset of the Bacteria Biotope 2019 Task [33] to evaluate our method. Bacteria Biotope 2019 is part of BioNLP Open Shared Task 2019 [34] and consists of several information extraction subtasks in the microbiology domain. The *BB-norm* subtask aims at normalizing Microorganism, Habitat, and Phenotype entities using taxonomies and ontologies. In our study, we focused on the normalization of Habitat and Phenotype entities. We chose to focus on these entities because they are expressed with noun and adjectival phrases that can take varied and complex forms. In contrast, microorganisms are mostly expressed in a codified way, based on proper nouns including a few orthographical variations. This type of named entities is typically easier to predict and classic rule-based methods usually yield high performance.

**Fig. 1:**
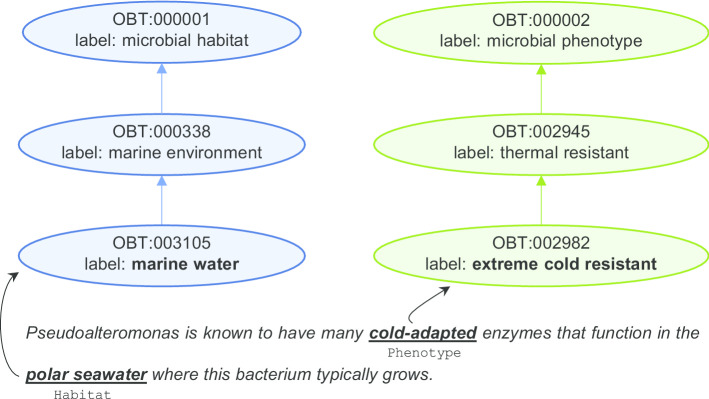
Normalization example for Habitat and Phenotype entities

Habitat and Phenotype entities have to be mapped to concepts of the OntoBiotope ontology [4]. The ontology consists of two main branches, the *microbial habitat* branch, which contains 3172 concepts and is used to normalize Habitat entities, and the *microbial phenotype* branch, which contains 429 concepts and is used to normalize Phenotype entities. The dataset is divided into a training, a development and a test set. Habitat and Phenotype entity annotations are provided in all sets and normalization annotations are provided in the training and development sets only. Figure 1 shows an example of the normalization task for Habitat and Phenotype entities. Each entity mention in the example text is mapped to an ontology concept.

Statistics of the dataset are given in Table 1. Its size is rather small, especially the Phenotype part of the dataset, in comparison with the size of the ontology. The main consequence is that some ontology concepts have few examples in the training data (if any). Indeed, the average number of examples per concept of the ontology is 0.6 with a standard deviation of 4.3 for habitats, and 1.2 with a standard deviation of 6.5 for phenotypes. The average number of examples per ontology concept occurring in the training data (training + development sets, as opposed to all ontology concepts) is 6.4 with a standard deviation of 12.3 for habitats, and 6.0 with a standard deviation of 13.5 for phenotypes, which is still very low for a classification task. Thus, the Bacteria Biotope corpus is a good example of the few-shot learning problem. Moreover, the task is also a good example of the zero-shot learning problem, since the proportion of concepts for which there is no example at all is quite high: 92.7% for habitats and 84.4% for phenotypes.

**Table 1:** Number of documents and annotations in the BB-norm corpus

	Train	Dev	Test
Documents	133	66	97
Habitat entities	1118	610	924
Phenotype entities	369	161	252
Habitat concepts	232	137	201
Phenotype concepts	67	44	49

### Overview of the method

Our approach draws on findings from previous work, in particular that of Ferré et al. [27], Deng et al. [29] and Limsopatham and Collier [30]. We observe from Ferré et al. [35] that the CONTES method is able to predict concepts that are relatively close to the target concepts in the semantic space, but has difficulty pinpointing the exact ones. We come to this conclusion by looking at the high non-strict performance score (where close matches are taken into account) obtained by CONTES in comparison with its lower strict performance score (where an exact match is required).

In contrast, shallow CNNs obtain a good overall score on datasets with strict evaluation [30], and a state-of-the-art score on datasets with non-strict evaluation [29], suggesting that they can make a significant number of exact predictions, but when they fail, the prediction is often semantically very far from the correct one in the ontology graph. Our hypothesis is that these two types of method are complementary and that it might be worth combining them.

Based on this hypothesis, we propose C-Norm, a synergistic ensemble method that combines two components: a single-layer feedforward neural network (SLFNN) for its ability to position the concepts well in the semantic space and a shallow convolutional neural network (CNN) for its ability to make exact predictions. We also implement a more standard sieve-based combination approach to compare it to C-Norm.

All components and combination methods use word vectors as input based on word embeddings trained on unlabeled corpora, and vectors of ontology concepts as output targets.

In the following, we first describe the construction of the word embeddings and concept vectors, then the SLFNN and shallow CNN components and the two combination methods.

### Preprocessing

All our methods are based on word embeddings from Word2Vec [18] and concept vectors from Ferré et al*.* [35, 36].

#### Word embeddings

We perform sentence and word segmentation on the corpus used to train Word2Vec, and to avoid out-of-vocabulary words, we apply the same word segmenter (see Availability of data and materials) on the labels of ontology concepts and on the BB-norm dataset. To obtain word embeddings adapted to the microbiology domain tackled by the BB-norm task, we use a dedicated corpus derived from PubMed, the same as in Ferré et al*.* [36]. In all these resources, we filtered stop-words (non-content words such as prepositions and determiners). All the initial embeddings have then been normalized to unit length.

#### Integrating ontological information in concept vectors

We use concept vectors as output targets of our neural network, following the work of Ferré et al*.* [27]. This strategy allows us to integrate knowledge from the ontology into our neural network-based method. More specifically, we encode hierarchical information (ancestor-child relations) in concept vectors. Concept vectors are built with the optimization proposed in Ferré et al*.* [35]. Each vector is built with a size equal to the number of concepts in the ontology, and each dimension is associated with a unique concept. The vector of a given concept has non-zero weights only for the dimension corresponding to the concept and for all dimensions corresponding to the ancestors of this concept (i.e. from the parents up to the root of the ontology). The weight is set to 1 for the dimension of the given concept. For the dimension of its ancestors, the weight decreases as we go up the hierarchical path in the ontology, that is, the farther away the ancestor is in the hierarchy the lower the weight is (the ontology root being the farthest ancestor, with the lowest non-zero weight). The formula for computing the weight is as follows:

$$\forall i,j \in \left\{ {1, ..., N} \right\}, c_{j}^{i} = w^{{d\left( {C^{i} ,C^{j} } \right)}}$$

where $N$ is the number of concepts in the ontology, ${c}_{j}^{i}$ is the *j*th weight of the vector of concept ${C}^{i}$, $w$ is a decay factor in [0,1], and $d({C}^{i},{C}^{j})$ is the number of steps of subsumption relation in the ontology hierarchy between ${C}^{i}$ and its ancestor ${C}^{j}$. The decay factor controls the importance given to ancestors. If the decay factor is set to zero, all representations become standard one-hot vectors, which allows the method to be used with non-hierarchical resources. If the decay factor is set to 1, all ancestors have the same weight as the current concept.

### Combining shallow neural networks

#### Component methods: single layer feedforward neural network and shallow CNN

**Fig. 2:**
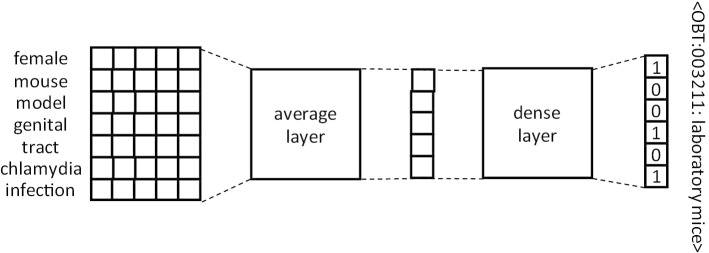
Architecture of the Single Layer Feedforward Neural Network. The input is the matrix of word embeddings of the non-stopword tokens of the mentions

*Single Layer Feedforward Neural Network (SLFNN)* Inspired by the CONTES method [27], the SLFNN aims to find a linear projection which globally aligns textual mention embeddings with the vectors of the concepts normalizing them. Embeddings of the words composing the mentions are averaged to obtain mention embeddings, which are then given to the dense layer of the SLFNN. We replaced the ordinary least squares method used in the original CONTES by a neural method to learn the projection. It allows the SLFNN to scale up to large training datasets and simplifies its combination with other neural architectures. The architecture of the SLFNN is shown in Fig. 2.

**Fig. 3:**
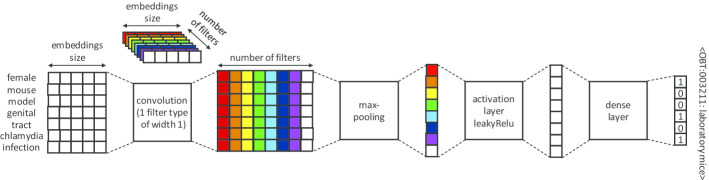
Architecture of the shallow CNN. The input is the matrix of word embeddings of the non-stopword tokens of the mentions

*Shallow CNN* the shallow CNN we used is inspired by Limsopatham and Collier [30], with no final softmax layer and with concept representations as in Ferré et al*.* [35]. As in other methods [27, 29] that do not use the usual one-hot vector representations for classes, classification is performed with a regression approach. It targets the concept vector nearest to the output vector computed for each mention, according to the cosine similarity. The objective remains the detection of a specific concept triggered by the presence of certain tokens in the input text, but the training also includes the additional constraint that the detection of a relevant concept must also trigger the detection of all its more general ancestors. This constraint stems from the fact that the output vectors are not standard one-hot vectors with only the weight of the specific concepts to predict set to 1, but also include non-zero weights for the ancestors, as described in Section *Integrating ontological information in concept vectors*. The intuition behind this constraint is that by learning parent–child proximities, the model will be able to minimize its prediction errors by predicting concepts that are close ancestors of the target concepts rather than concepts far away in the ontological graph. Figure 3 shows the architecture of the shallow CNN.

#### Combination methods: Sieve and C-Norm

**Fig. 4:**
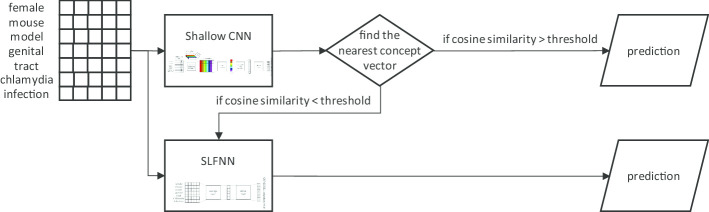
Architecture of our Sieve method using our SLFNN and shallow CNN methods. The inputs are the matrix of word embeddings of the tokens of the mentions

*Sieve* The SLFNN and CNN components each produce a prediction of a concept and a cosine similarity score between a textual mention and its predicted concept (i.e. the concept with the nearest vector). We hypothesize that this score can act as a confidence score and that we could compute an optimal threshold to select predictions having the highest probability of being correct. Thus, we designed a sieve-based method that first uses the shallow CNN to make predictions for all mentions, keeps only the predictions with a score above the chosen threshold, and then gives the remaining mentions to the SFLNN component. This process is illustrated in Fig. 4. Among the two components, we empirically chose the shallow CNN as the first component to produce initial predictions.

**Fig. 5:**
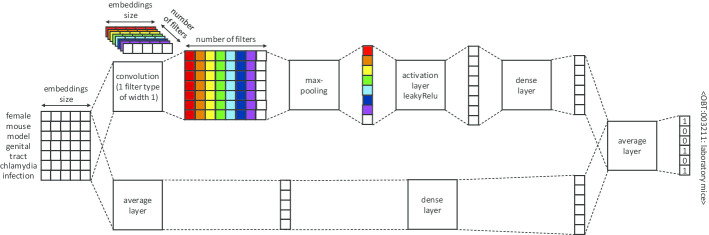
The C-Norm architecture combining the SLFNN and shallow CNN components. The input is the matrix of word embeddings of the tokens of the mentions

*C-Norm* The Sieve method includes a new hyperparameter to be manually set: the confidence threshold. However, the optimal value of the threshold may be highly variable depending on the task and it may be difficult to determine without annotated gold standards. Moreover, the Sieve method is a rigid way to combine the SLFNN and the Shallow CNN. The C-Norm method aims to combine more the two methods more efficiently. We parallelize both methods within a single neural architecture that combines their outputs with an averaging layer. Averaging outputs is a common ensemble method that yields good performance. We chose to do this in an end-to-end way in order to allow the combination step (the averaging layer) to give feedback to the individual components during training, as opposed to a static averaging of the outputs after independent training of the components. The architecture can then directly learn a smoother way to combine the contributions of each component. Figure 5 shows the architecture of C-Norm with the interaction of the two components.

### Experimental setting

#### Training sets

To offset the small size of the manually annotated datasets, we followed a weak supervision strategy to increase the size of the training set with non-manually annotated examples. We used the labels of the ontology concepts as examples of normalized mentions, i.e. each concept label is handled as an entity mention normalized with that same concept. This strategy has shown good results in previous work on the 2016 edition of the Bacteria Biotope task [36].

When tuning the models, we trained on the manually annotated training set augmented with ontology labels and used the development set as validation data. When evaluating on the test set, we also added the development set to the training data. Thus, our approach combines standard supervision (using the manually annotated BB-norm corpus) with weak supervision (using the ontology concept labels as training examples).

#### Hyperparameter setting

*Word embeddings* We used embeddings computed by Word2Vec with the same training corpus and the same hyperparameters as those described in Ferré et al*.* [36] for the CONTES method, except for the embedding size which we set to a smaller value of 200 (compared to 1000) which was computationally more efficient while still yielding high performance.

*Concept vectors* To smooth the non-zero values of ancestor dimensions and compared to the initial CONTES method which had an equivalent decay factor of 1.0 (all non-zero weight to 1), a subsequent study [35] showed that a factor of 0.6 may be a better choice to improve simultaneously strict and nonstrict performance scores. Thus, we chose this value for all our experiments.

*Neural network hyperparameters* We tuned the hyperparameters of the models on the development set of the BB-norm corpus. Most hyperparameters were chosen empirically by training multiple runs of the models for Habitat normalization and were re-used for models normalizing Phenotypes. We did so because the Phenotype part of the dataset is much smaller than the Habitat part and thus it is harder to observe clear tendencies on this dataset. For all models, we chose the Nadam optimizer [37] and the log_cosh loss function, after experimenting with a number of options—Stochastic Gradient Descent, Adam [38], Adadelta [38], Amsgrad [39] and Nadam [37] for the optimizer and mse, cos, huber, mae, and log_cosh for the loss function. The advantage of the log_cosh function, besides showing good performance (either the highest or similar performance compared to other loss functions), was to yield more stable results across repeated runs. For all models, the number of epochs was empirically chosen by evaluating the performance at different epochs and stopping when performance did not visibly improve anymore.

We list below the hyperparameters chosen for each method:

- *SLFNN* We set the number of epochs to 50 for Habitat models and 100 for Phenotype models.
- *Shallow CNN* We set the number of filters to the size of the ontology (i.e., 3172 for Habitat models and 429 for Phenotype models). We selected a single filter size of 1, after experimenting with a few single sizes (1 to 5) as well as with some combinations of sizes based on previous work by Limsopatham and Collier [30] (specifically, sizes 3, 4 and 5]). We chose the leakyReLU activation function [40] directly at the end of the maxpool layer, after experimenting with leakyReLU, ReLU, tanh, and softsign. We set the number of epochs to 150 for Habitat models and 50 for Phenotype models.
- *Sieve* We set the cosine similarity threshold of the Sieve method to 0.4, after experimenting with values ranging from 0.9 to 0.1 (in 0.1 increments). We observed that 0.3 and 0.4 both worked best compared to other values (approximately 6 points higher than the worst threshold values), and chose the higher value between the two.
- *C-Norm* We set the number of epochs to 200 for Habitat models and 30 for Phenotype models. The other hyperparameters are those of the Shallow CNN component (activation function, filter size, filter number).

#### Evaluation strategy

The official evaluation tool of the BB-norm task [33] was used to compute performance scores on the test set of the task. We also report results on the development set, including an evaluation of the performance of all components and combination methods, and analyses of the effect of using weakly supervised data and of using ontological information. More precisely, we look at the performance of our best-performing method with versu without using the ontology labels as training examples, and at its performance with different values of the decay factor (used to build concept vectors), including a factor of 0 which is equivalent to generating one-hot concept vectors with no ontological information.

The metrics of the BB-norm shared task are a similarity score and a strict exact match score. The strict exact match score is equal to 1.0 if the predicted concept is equal to the reference concept, 0.0 otherwise. This score can be relatively harsh for tasks where the references are semantically related (e.g. two semantically close subsumed concepts). Therefore, the BB-norm task uses a smoother similarity score based on the semantic distance between two concepts in an ontology, as defined by Wang et al*.* [41]. For a prediction, this similarity score is calculated between the reference concept and the predicted concept, having the value of 1.0 if both concepts are equal, and tending towards 0.0 if the concepts are farther in the hierarchical graph of the ontology. The overall score of a dataset prediction is the average of all scores for each mention.

We compared our approach to those of the official participants of the BB-norm 2019 shared task and to the baseline provided by the task organizers. The baseline is a simple rule-based method that performs exact matching between lemmatized entity mentions and ontology concept labels. Most approaches from the participants use word embeddings and machine-learning, but not all of them. The PADIA BacReader team [29] combined a voting ensemble of shallow CNNs with a rule-based baseline in a sieve way. The BLAIR GMU team [42] used a machine learning and embedding-based method. The BOUN-ISIK team [43] used a static word embedding-based method integrating syntactic information. The AmritaCen Healthcare team also participated but did not publish the details of their method.

We also included in the comparison two machine learning and embedding-based methods that obtained state-of-the-art results on a previous version of the Bacteria Biotope task in 2016 [44]: the CONTES method and the sieve-based HONOR method [36]. We used the best hyperparameters of those two methods as described in Ferré et al*.* [35, 36]: we trained the models on the training and development sets of the task and on the ontology labels, and used a decay factor of 0.6 to build the concept vectors and an embedding size of 1000.

## Results

In this section, we report results obtained on the development set by the neural components and combination methods. We analyze the effect of using weakly supervised data and ontological knowledge. Then we show the performance of the C-Norm method on the test set and compare it with existing approaches.

### Experiments on the development set

#### Performance of the different methods

Table 2 shows performance obtained on the BB-norm development sets for each neural network component and each combination architecture. Results are averaged over 10 runs to account for variations in the initialization of the neural networks and standard deviation is provided.

**Table 2:** Performance of all methods on the BB-norm development set (mean with standard deviation)

	BB-norm habitats		BB-norm phenotypes	
	Wang score	Strict score	Wang score	Strict score
SLFNN	0.654 ± 0.003	0.325 ± 0.004	0.814 ± 0.013	0.537 ± 0.011
S-CNN	0.696 ± 0.003	0.510 ± 0.007	0.782 ± 0.005	0.501 ± 0.013
Sieve (threshold = 0.4)	0.725 ± 0.003	0.508 ± 0.005	0.807 ± 0.008	0.527 ± 0.009
C-Norm	*0.819* ± 0.004	*0.633* ± 0.009	*0.854* ± 0.011	*0.620* ± 0.024

The highest scores are in italics

The shallow CNN component performed better than the single-layer feedforward NN for Habitats. The SLFNN performed almost 20 points lower in terms of strict score, but its similarity score remains quite high (0.654 vs. 0.696). This suggests that the two methods are complementary: the S-CNN does better at pinpointing the exact concept, but the SLFNN is able to position the mentions quite well in the concept vector space (which explains the high Wang score). On the Phenotype normalization task, the two methods obtained similar results measured by both scores, the SLFNN performing slightly higher, although the very small size of the Phenotype dataset makes it hard to draw any conclusion.

The Sieve combination architecture performed slightly better than both components for Habitat entities (in terms of Wang score), and similarly to the SLFNN for Phenotype entities.

The C-Norm method outperformed all methods by at least 10 points for both types of score on habitat normalization, and by 4 and 7 points respectively for each score on phenotype normalization. It demonstrates the benefit of combining the SLFNN and S-CNN components as well as of using a smoother integration method than a sieve-based approach.

In light of these results on the development set, we did not evaluate all methods on the test set, but focused on the C-Norm method and compared it to existing approaches.

#### Effect of using weakly supervised data

Table 3 shows the performance of the C-Norm method for Habitat entities on the development set using only the manually annotated training set (standard supervision) and using both the manual annotations and the ontology labels as training data (standard + weak supervision). We see that the gain in performance is substantial, i.e. more than 10 points for both types of score, thus demonstrating the benefit of adding ontology labels to the training set.

**Table 3:** Performance of C-Norm for Habitats on the development set, using standard supervision vs. standard + weak supervision

	Wang score	Strict score
C-Norm *standard*	0.698 ± 0.003	0.473 ± 0.004
C-Norm *standard* + *weak*	0.819 ± 0.004	0.633 ± 0.009

The type of supervision used is in italics

#### Effect of using ontological knowledge

**Fig. 6:**
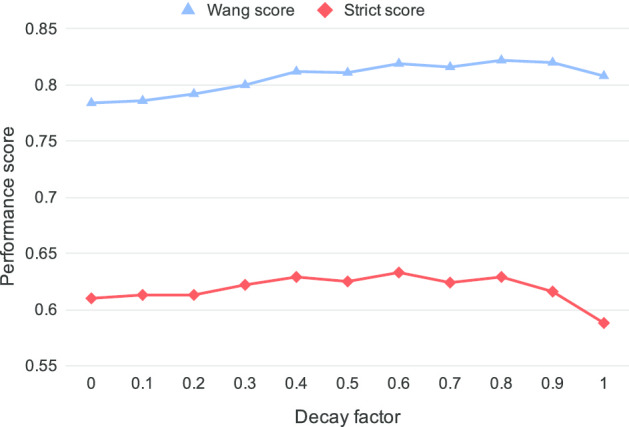
C-Norm performance scores for Habitats using different decay factors

Figure 6 plots the performance of C-Norm for Habitat entities using different values of the decay factor (ranging from 0 to 1, with 0.1 increments). A value of 0 means that the hierarchical relation between parent and child concepts is not taken into account in the output vector, thus no ontological knowledge is used. We can see that using the hierarchical information (i.e., decay factors > 0) consistently increases performance. The choice of the value of the decay factor is not clear-cut, as values from 0.4 to 0.8 all work well. However, a value of 0.6 yields a high similarity score as well as the highest strict score, and so seems a reasonable choice. Compared to not integrating ontological knowledge, using the 0.6 decay factor increases the Wang score by 3.5 points and the strict score by 2.3 points.

#### Evaluation results

Table 4 gives a summary of the characteristics of the methods evaluated on the test set of the BB-norm task, including the baseline, methods from official teams who participated in the BB-norm shared task (AmritaCen Healthcare, BOUN-ISIK, BLAIR GMU and PADIA BacReader), methods having obtained high performance on a similar dataset (CONTES and HONOR), and our best performing method C-Norm.

**Table 4:** Characteristics of the methods

	Embedding-based	ML-based	Rule-based
Baseline	No	No	Yes
AmritaCen Healthcare	n/a	n/a	n/a
BOUN-ISIK	Yes	No	Yes
BLAIR GMU	Yes	Yes	No
PADIA BacReader	Yes	Yes	Yes
CONTES	Yes	Yes	No
HONOR	Yes	Yes	Yes
C-Norm	Yes	Yes	No

Performance of the methods is shown in Table 5. C-Norm significantly outperforms all existing methods according to both measures, non-strict evaluation (Wang similarity score) and strict evaluation (strict exact score): respectively + 4 and + 7 points compared to the best approach (HONOR) for Habitat normalization, and + 9 points (for both scores) compared to the best approach (CONTES) for Phenotype normalization.

**Table 5:** Performance on the test set (95% CI = 95% confidence interval)

	Habitats		Phenotypes	
	Wang [95% CI]	Strict [95% CI]	Wang [95% CI]	Strict [95% CI]
Baseline	0.559 [0.543, 0.576]	0.224 [0.199, 0.250]	0.581 [0.559, 0.604]	0.091 [0.056, 0.127]
AmritaCen	0.522 [0.497, 0.548]	0.347 [0.314, 0.376]	0.646 [0.595, 0.698]	0.512 [0.448, 0.571]
BOUN-ISIK	0.687 [0.667, 0.710]	0.428 [0.395, 0.459]	0.566 [0.520, 0.610]	0.315 [0.259, 0.373]
BLAIR GMU	0.615 [0.596, 0.632]	0.211 [0.185, 0.237]	0.646 [0.607, 0.685]	0.313 [0.254, 0.373]
PADIA	0.684 [0.661, 0.709]	0.488 [0.456, 0.519]	0.758 [0.716, 0.803]	0.618 [0.556, 0.676]
CONTES	0.715 [0.694, 0.736]	0.500 [0.467, 0.529]	0.799 [0.760, 0.835]	0.616 [0.553, 0.675]
HONOR	0.737 [0.716, 0.759]	0.531 [0.499, 0.563]	0.778 [0.738, 0.814]	0.578 [0.519, 0.640]
C-Norm	*0.777* [0.755, 0.797]	*0.604* [0.574, 0.635]	*0.881* [0.855, 0.907]	*0.700* [0.643, 0.755]

The highest scores are in italics

## Discussion

C-Norm significantly outperforms other methods on the two datasets of the 2019 BB-norm task. An advantage of the C-Norm method is also that it is domain-independent, in contrast with methods such as HONOR which obtained good results but used domain-specific rules.

The C-Norm ensemble method is able to improve upon the performance of the individual SLFNN and S-CNN components. We performed an error analysis on predictions made on the development set by the best run of our methods. Out of the 610 mentions in this set, 382 mentions (62.6%) have been correctly normalized by C-Norm. Compared to C-Norm, the SLFNN has only correctly normalized 194 mentions (31.8%), the S-CNN only 308 mentions (50.5%) and the union of the correct predictions of both components results in 342 mentions (56.1%). C-Norm correctly normalized 91 mentions (15%) which have been incorrectly normalized by the SLFNN and the S-CNN. It shows that the particular combination of the SLFNN and the S-CNN in C-Norm is able to take advantage of their complementarity. Nevertheless, the SLFNN has correctly normalized 12 mentions (2%) which have not been correctly normalized by C-Norm and 48 mentions (7.9%) for the S-CNN. However most of these newly introduced errors (85%) are assigned relatively weak penalties by the Wang similarity score compared to errors from the SLFNN and the SCNN, meaning that they are not severe errors (see the following paragraph for a typology of errors).

Despite the high performance of C-Norm, few-shot normalization is still challenging and there is room for improvement. The error analysis on the development set revealed that 228 mentions (37.4%) out of 610 were incorrectly normalized. We classified errors into several types:

- *Partially correct normalization* 40 mentions (17.5% of the errors) have only been partially correctly normalized. Indeed, the BB-norm dataset includes mentions that have to be normalized with more than one concept (e.g. the mention “healthy adult” has to be normalized with both <OBT:002712: healthy person> and <OBT:003245: adult human>). Existing methods, including C-Norm, typically only predict one concept per mention.
- *Overgeneralization* 64 mentions (28.1% of the errors) have been normalized by a concept more general (i.e. higher in the is_a hierarchy graph) than an exact one, and the predicted concepts are at a maximum of 5 concepts above the exact concept (e.g. the “scimudin cheese” mention should have been normalized by the concept <OBT:003522: Scimudin>, and has been normalized by <OBT:001480: cheese>, and <OBT:003522: Scimudin> is_a <OBT:003492: mould ripened cheese>, which is_a <OBT:003459: soft cheese>, which is_a <OBT:003428: ripened cheese>, which is_a <OBT:003381: fermented cheese>, which is_a < BT:001480: cheese>).
- *Overspecification* On the contrary, 36 mentions (15.8% of the errors) have been normalized by a concept more specific (i.e. lower in the is_a hierarchy graph) than an exact one, and the predicted concepts are at a maximum of 2 concepts below the exact concept (e.g. the mention “ulcer” has been normalized by <OBT:001533: duodenal ulcer>, and should have been normalized by <OBT:000933: ulcer>, and <OBT:001533: duodenal ulcer> is_a <OBT:001248: peptic ulcer>, which is_a <OBT:000933: ulcer>).
- *Other* The remaining errors (88 mentions, 38.6% of the errors) corresponds to mentions normalized with concepts that are not in the hierarchical path of their correct concept, i.e., they were normalized neither with an ancestor nor with a child.

We consider the first three types of errors less serious than the last one. They are assigned weaker penalties by the Wang similarity score. At an equivalent distance in the graph between predicted and correct concept, the last type of error is more penalized than an overgeneralization/specification error by the similarity measure. Moreover, these errors are globally the worst in terms of distance to the correct concept: they range from 2 to 16, with a median at 8. Thus, we estimate that this type of error should be addressed as a priority in future work. We analyzed them in detail and found that, out of these 88 mentions, there were two main sources of errors:

- Syntactic structure of entity mentions (31 mentions, 35.2%): our method has sometimes trouble determining the core meaning of the multi-word mentions. For instance, the mention “*chicken nugget processing plant*” has been automatically annotated with the concept <OBT:002729: *nugget*>, rather than with <OBT:002129: *meat industry*>, and the mention “*hyperimmune mouse sera*” with <OBT:002727: *mouse*> rather than with <OBT:000524: *blood serum*>. Taking into account syntactic information such as the identification of the syntactic head of entity mentions could contribute to improve performance in these cases.
- *Ambiguities (16 mentions, 18.2%)* Our method does not take into account the context of entity mentions and thus does not handle well ambiguities such as polysemic words. For instance, the word “*malt*” can either mean “*dried germinated cereal grains*” (represented by the concept <OBT:003215: *malt*>), or be the acronym of “*mucosa-associated lymphoid tissue*” (which is a <OBT:000334: *lymphatic system part*>). Another example is the one-word mention “*chicken*”, which is often annotated with the concept <OBT:003314: *chicken*>, but should sometimes be annotated with the concept <OBT:002394: *chicken meat*>, depending on the context.

We identified potential solutions to address these limitations. A way to take into account syntactic information would be to use graph-neural networks [45] to represent graph nodes, such as words in a syntactic dependency tree [46]. This kind of networks could be used as a bottom layer for C-Norm, enabling the method to give more weight to syntactic heads compared to modifiers when computing an intermediate representation for each mention. To take into account the context of a mention, at least the intra-sentence context, a first step could be to replace Word2Vec word embeddings with context-aware word embeddings such as those computed by ELMo [19] or BERT [20]. We plan to investigate these interesting directions in future work.

The weak supervision strategy results in a large increase in performance (+ 12.1 points for Wang score and + 16 for strict). We hypothesize that there are three main explanations for this increase:

- *An augmentation of the training data* Few-shot classification methods can primarily take advantage of data augmentation, and adding labels almost triples the total number of examples.
- *A better coverage of the training data* All the concepts in the ontology have at least one label, so the new training data covers 100% of the ontology concepts. It really differs from the manually annotated examples, which cover only 7.3% of the ontology concepts.
- *An ontology well-suited for natural language processing* The OntoBiotope ontology has been built from expert analysis of microbiological publications. This implies that the labels are terms which are close to those found in texts. However, this feature is dependent on the task and the ontology, and will not necessarily generalize well to other contexts. For instance, in the Social Media Mining for Health Applications (SMM4H) shared task [47] where adverse drug reaction mentions in tweets are normalized with concepts from the MeDRA terminology, the language used in tweets can be quite different from term labels (e.g. “head is killing me” normalized with the concept <MEDDRA:10019211: headache>).

C-Norm has been evaluated on two normalization tasks belonging to the same dataset from a specialized domain (i.e. scientific literature in microbiology). To demonstrate the actual adaptability of the method to other domains, evaluations on others tasks should be conducted in the future, such as the Social Media Mining for Health Applications (SMM4H) dataset [47] or the TAC Adverse Drug Reaction Extraction from Drug Labels dataset [48].

## Conclusions

C-Norm is a new neural method which synergistically integrates several strategies to handle few-shot normalization tasks: weak supervision, ontological knowledge integration and distributional semantics. C-Norm is built in an ensemble averaging way, which learns to dynamically combine predictions from two neural components with complementary results: a shallow CNN and a single layer feedforward neural network. These components are representative of two recent types of approach that have shown good performance in entity normalization in specialized domains. Our results show that the choice of components and the method to combine them is the source of a significant gain in the predictive capacity of algorithms, greatly outperforming other methods on the Bacteria Biotope datasets of BioNLP-OST 2019, while keeping an overall relatively shallow algorithm.

## Data Availability

The datasets analyzed during the current study are available at: https://sites.google.com/view/bb-2019/dataset. The code for the neural methods described in the current study is available in the following repository: https://github.com/ArnaudFerre/C-Norm, under the license APACHE LICENSE, VERSION 2.0. The WosMig word segmenter used to preprocess the corpora is documented at https://bibliome.github.io/alvisnlp/reference/module/WoSMig, and is available as part of the AlvisNLP/ML pipeline: https://github.com/Bibliome/alvisnlp under the license APACHE LICENSE, VERSION 2.0. The tool used to evaluate performance is available online at: http://bibliome.jouy.inra.fr/demo/BioNLP-OST-2019-Evaluation/index.html

## Abbreviations

BB-norm: Bacteria Biotope normalization
BioNLP-OST: BioNLP Open Shared Tasks
CI: Confidence interval
CNN: Convolutional neural network
ML: Machine learning
ReLU: Rectified linear unit
S-CNN: Shallow convolutional neural network
SLFNN: Single layer feedforward neural network
SMM4H: Social Media Mining for Health Applications
TAC: Text analysis conference
TF-IDF: Term frequency-inverse document frequency
